# Tumors in the setting of dupilumab use: A review of the literature^⁎^

**DOI:** 10.1016/j.waojou.2024.101006

**Published:** 2024-12-11

**Authors:** Shumeng Guo, Liangchun Wang, Dingfang Bu, Fengjie Liu

**Affiliations:** a Department of Dermatology, Sun Yat-sen Memorial Hospital, Sun Yat-sen University, China; b Department of Dermatology, Peking University First Hospital, Beijing, China

**Keywords:** Dupilumab, Atopic dermatitis, Tumor, Cutaneous T-cell lymphomas

## Abstract

Dupilumab is the first monoclonal antibody approved for treating moderate-to-severe atopic dermatitis (AD) and has significantly improved the quality of life of AD patients. However, the safety of dupilumab is yet unclear in the context of cancer. Therefore, we aimed to investigate the safety of dupilumab and its relationship with the progression and occurrence of tumors. By reviewing relevant medical records of 90 patients who had pre-existing tumors before dupilumab treatment or presented new tumors after dupilumab treatment, we found that dupilumab probably had no significant negative effects on most tumors, but several patients with Cutaneous T-cell lymphomas (CTCLs) had relatively unfavorable outcomes during dupilumab treatment. Besides, CTCLs and lymphomas accounted for the majority of patients who presented new tumors after dupilumab treatment. Several patients were first diagnosed with presumed AD and probably were the presentations of CTCL at an early stage, and they developed typical CTCL symptoms after dupilumab treatment. Finally we came to the conclusion that dupilumab is safe for most patients with cancer. However, the effect of dupilumab on CTCLs is disputable. The use of dupilumab requires individual evaluation and closely monitored. When the efficacy is poor, re-evaluation of the diagnosis, especially of CTCLs and related diseases, is necessary.

## Introduction

Dupilumab is a fully human monoclonal antibody against the interleukin (IL)-4 receptor alpha subunit (IL-4Rα). Binding of the monoclonal antibody to the IL-4Rα inhibits the signaling of IL-4 and IL-13, the 2 major cytokines secreted by CD4^+^ T-helper 2 (Th2) cells.^1^ Dupilumab has been approved for the treatment of moderate-to-severe atopic dermatitis (AD) not adequately controlled by topical therapies and has become the first monoclonal antibody for the treatment of AD.^1^

Cutaneous T-cell lymphomas (CTCLs) are characterized by mature CD4^+^ T-helper cells that are remarkably Th2-biased with strong inhibition of Th1 responses.^2^^,^^3^ Blocking IL-4/IL-13 signaling pathways by anti-IL-4 neutralizing antibody reduces the proliferation of mycosis fungoides (MF) cells.^2^ IL-4 and IL-13 are the major cytokines transforming the tumor-associated macrophages (TAMs) to M2 macrophages that promote cancer progression and treatment resistance, and dupilumab reduces the pro-tumor phenotype of M2 macrophages.^4^ However, previous studies have shown that IgG4 is highly expressed in various types of tumor tissues, such as pancreatic cancer,^5^ gastric cancer,^6^ and others. IgG4 reduces the expression of CD206, CD163, and CD14 on the surface of M2 macrophages, increases the production of CCL-1, IL-10, and IL-6, induces the M2b-like macrophage phenotype, which impairs the tumor cell phagocytosis function and the function of anti-cancer effector cells.^7^ Therefore, dupilumab, a fully human IgG4 monoclonal antibody, may induce macrophage polarization to M2b, mediating tumor tolerance and ultimately leading to cancer progression. Previous studies have reported that dupilumab may cause the worsening of existing tumors prior to the antibody therapy or may drive the appearance of typical tumors, in AD or refractory pruritus patients during or after dupilumab treatment. These tumors include CTCLs, other skin tumors, hematologic tumors, and solid tumors. For example, dupilumab treatment unmasked the atypical lymphoid infiltrates or MF in patients with refractory presumed AD and pruritus.^8^ In this article, we collected and analyzed the relevant cases reported in the literature to explore the safety of dupilumab treatment for AD or refractory pruritus and the possible mechanisms of dupilumab on tumors.

## Methods

### Search strategy

**Fig. 1:**
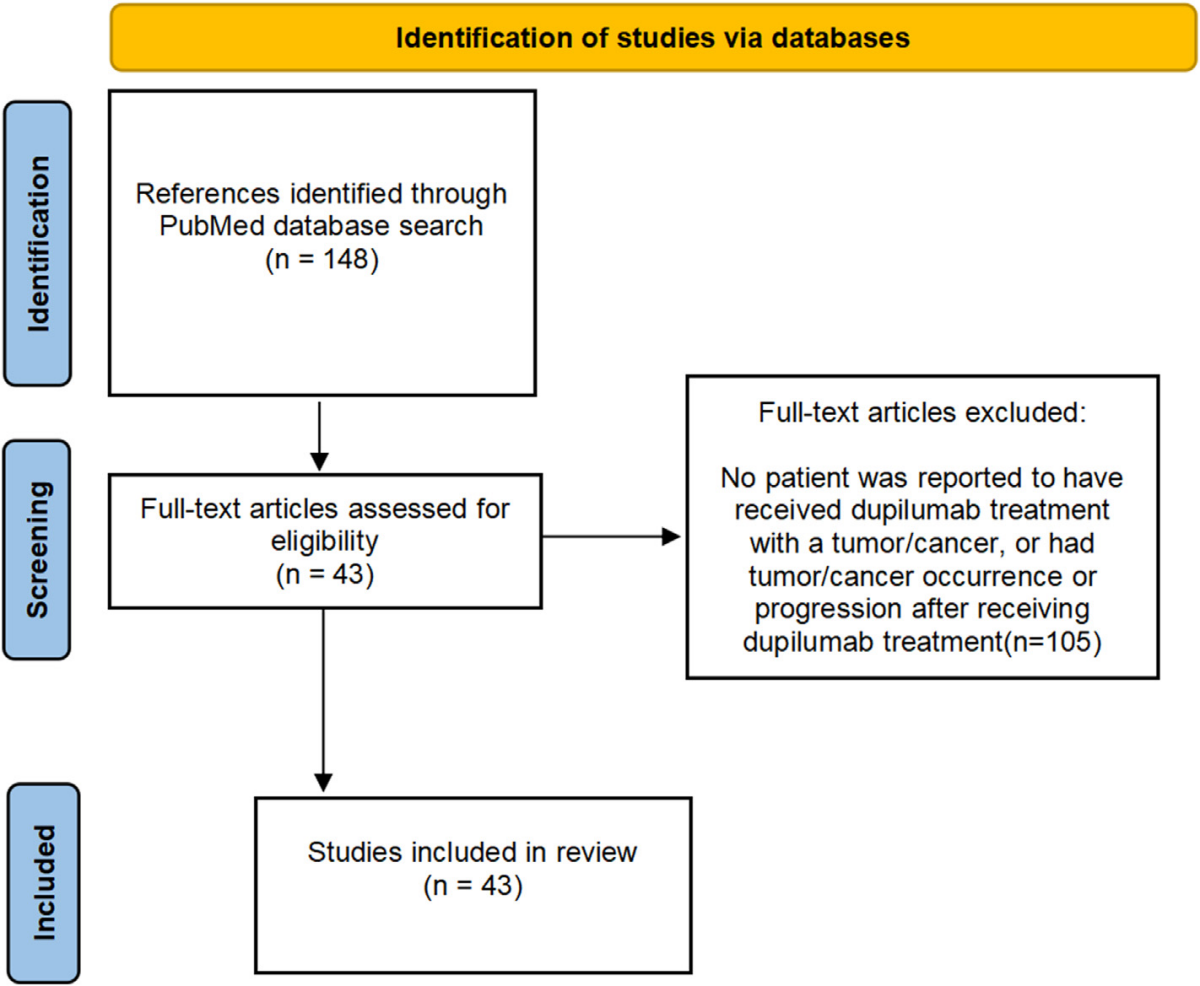
PRISMA flow diagram of systematic review

A systematic search in the PubMed database was performed following the Preferred Reporting Items for Systematic Reviews and Meta-Analyses (PRISMA) guidelines (Fig. 1). We used the keywords “dupilumab AND cancer” and “dupilumab AND tumor” without limitation of article type to search the English publications in PubMed from 2017, the beginning of dupilumab for clinical use, until August 2024. A total of 148 full-text and eligible articles were retrieved, of which 43 articles were analyzed in this study after excluding the eligible articles without reported case(s) (n = 105).

### Patients

A total of 90 patients, including AD, presumed AD, and other diseases with refractory pruritus, were reported in the 43 articles. Their demographics, preliminary diagnosis and treatment, dupilumab treatment method, the tumor diagnosed before or after dupilumab treatment, tumor type, TNM (tumor, node, and manifestation) classification and stage, changes of tumor and skin lesions after dupilumab treatment, and outcome of the patient were extracted from the 43 articles.

### Dupilumab treatment regimen and assessment

A 600 mg dupilumab loading dose followed by 300 mg dupilumab every 2 weeks was used in most cases. The therapeutic effect was evaluated mainly according to the changes in skin rash, pruritus score, and quality of life index. We paid special attention to the occurrence of tumors and changes in tumor progression to evaluate the effect of dupilumab treatment on tumors.

## Results

The summary statistics of 90 patients are shown in Table 1, and the specific clinical characteristics are shown in Table S1. They were aged 22–82 years old. Except for 7 patients whose gender was not specified, 50 patients were males and 33 were females, with a male-to-female ratio of 1.52:1. The course of dupilumab treatment ranged from 1 injection to several years. It is important to note that all patients had concomitant tumors or newly developed tumors after the use of dupilumab.

**Table 1:** Demographic characteristics and changes of disease in patients with concomitant or newly emerging tumors treated with dupilumab.

Demographic characteristic	Patients, No. (%)
Male sex	50 (55.6)
Female sex	33 (36.7)
Unknown sex	7 (7.8)
Age	22–82 years old
Dupilumab treatment duration	Once to 30 months
**Tumor characteristic**	
Pre-exist before dupilumab treatment	62
With primary tumors	57 (63.3)
CTCL misdiagnosed as AD	3 (3.3)
With primary solid tumors, new CTCLs	2 (2.2)
Present after dupilumab treatment	30
With new tumors	28 (31.1)
With primary solid tumors, new CTCLs	2 (2.2)
**Tumor type**	
CTCL	34 (37.8)
Other skin tumors	9 (10)
Hematological	24 (26.7)
Solid tumors	26 (28.9)
**Tumor changes**	
Stable	14 (15.6)
Progression	10 (11.1)
Response	13 (14.4)
Not available	53 (58.9)
**Primary dermatological changes**	
No response	24 (26.7)
Response	40 (44.4)
Not available	26 (28.9)

AD, atopic dermatitis; CTCL, Cutaneous T-cell lymphoma.

### Patients with tumors before dupilumab treatment

A total of 62 patients had the tumors before dupilumab treatment.^8^, ^9^, ^10^, ^11^, ^12^, ^13^, ^14^, ^15^, ^16^, ^17^, ^18^, ^19^, ^20^, ^21^, ^22^, ^23^, ^24^, ^25^, ^26^, ^27^, ^28^, ^29^, ^30^, ^31^, ^32^, ^33^, ^34^, ^35^, ^36^ Most patients showed improvement in AD rash and pruritus, and only 8 patients showed aggravation of rash and pruritus.^8^^,^^13^^,^^37^ The changes in tumors after dupilumab treatment are listed in Table 2. It seems that dupilumab has no significant negative effects on these tumors, except that more CTCL patients under dupilumab treatment are required to be observed to draw a conclusion.

**Table 2:** Patients with pre-existing tumors and changes in the tumor after dupilumab treatment.

Tumor	No.	Stable	Death or progression	Partial response	Remission or very good response	Changes not available
Skin (CTCL: MF, SS, CTCL-NOS)	10		5 (case 1,5,6,7,25) [8,22]	3 (case 19,21,26) [12,21,29]		2 (case 33,34) [13,17]
Skin (melanoma, squamous cell carcinoma, angiosarcoma)	9	3 (case 39,40,41) [15,26,32]		2 (case 38,42) [15,30]	2 (case 35,36) [35]	2 (case 33,37) [17]
Hematological (multiple myeloma, lymphoma)	21	1 (case 53) [31]	3 (case 54,59,61) [19]	2 (case 38,62) [15,20]	2 (case 52,60) [18,20]	13 (case 43,44,45,46,47,48,49,50,51,55,56,57,58) [10,19,24,25,33]
Solid tumors	25	10 (case 78,79,82,83,84,85,86,87,88,90) [16,17,27.28,34]	1 (case 89) [15]	2 (case 42,76) [23,30]	2 (case 77,80) [11,34]	10 (case 15,31,66,67,68,69,70,71,72,75) [9,10,14,26,36]

P.s.: case 15 and case 31 were originally diagnosed with solid tumors and presented CTCL after dupilumab treatment. Case 33,38 and 42 had overlapping multiple tumor types, so they were counted twice.

### Patients present tumors after dupilumab treatment

A total of 30 patients presented tumors after dupilumab treatment (1 patient subjected to only 1 injection of dupilumab is excluded from the analysis).^8^^,^^10^^,^^14^^,^^21^^,^^36^, ^37^, ^38^, ^39^, ^40^, ^41^, ^42^, ^43^, ^44^, ^45^, ^46^, ^47^, ^48^, ^49^ Interestingly, 23 of the 29 patients developed CTCLs. Most authors believed that the pre-existing malignant T-cell clone may overgrow in a changed immune microenvironment. For example, some patients were first diagnosed with presumed AD (cases 1, 2, 3, 4, 16) and actually were the presentations of CTCL at an early stage; they developed typical CTCL symptoms after dupilumab treatment.^8^^,^^49^ Therefore, careful examination of the refractory patients with atypical AD lesions to identify the possibility of concomitant tumors, especially CTCLs, before dupilumab treatment is recommended. On the other hand, in some patients with long-standing AD, eczematous or psoriasiform lesions confirmed by multiple biopsies, CTCLs occurred following dupilumab treatment (cases 24,27,28,29,30,63,64).^36^^,^^41^^,^^42^^,^^46^^,^^48^

Three patients (cases 73,74,81) developed testicular tumors, embryonic cancer, or bladder cancer after dupilumab treatment.^10^^,^^39^ Whether these tumors were coincidental events or a specific correlation with the inhibition of IL-4/IL-13 is uncertain.

## Discussion

### Roles of IL4/13 in CTCL

Type 2 immunity, illustrated by T helper 2 lymphocytes (Th2) and downstream cytokines (IL-4, IL-13, IL-31) as well as group 2 innate lymphoid cells (ILC2), is important in host defense and wound healing.^50^ In CTCL, the expression of STAT4, an important transcription factor of Th1 lymphocyte subsets, is upregulated in the early stage. However, with the development of the disease, the expression of signal transducer and activator of transcription 4 (STAT4) is usually lost, leading to the switch from Th1 to Th2, causing cancer progression and immunosuppression, which is associated with worse clinical prognosis.^51^ The 2 most common CTCLs, advanced MF and SS, are often associated with eosinophilia and high IgE levels.^52^ IL-4 and IL-13 are the main cytokines that drive the Th2 response and inhibit Th1/Th17 differentiation.^53^^,^^54^ They are also important growth factors in primary cutaneous lymphoma, where IL-13 acts on tumor lymphocytes in an autocrine manner.^2^ Both are involved in stimulating B-cell differentiation, IgE production, eosinophilic growth, and aggregation^53^, ^54^, ^55^ and have been confirmed as irritants of chronic pruritus.^53^^,^^54^ Therefore, inhibition of the IL-4/13 pathway can theoretically improve the clinical symptoms of CTCL.

It has been reported that IL-13 is an autocrine factor in CTCL. CTCL cells produce IL13 and express IL13 receptors, which can induce the growth of CTCL cells. Moreover, pSTAT6 was highly expressed in CTCL lesions, implying the activation of the IL4/IL13 pathway. It was confirmed that blocking IL-4 and IL-13 had a synergistic effect on inhibiting the growth of CTCL cells. Interestingly, blocking IL-13Rα2 revealed an even stronger inhibition of tumor growth, considering that IL-13 binds to IL13Rα2 more strongly than IL13Rα1.^2^^,^^56^ Therefore, blocking the heterodimer formed by IL-4Rα and IL-13Rα1 may increase the binding of IL-13 to the IL-13Rα2 site. Effectively increasing the IL-13 shunt in the tumorigenic pathway may achieve a tumor promotion effect.

### Roles of IL-4/13 in other tumors

TAMs are abundant tumor-associated macrophages in the tumor microenvironment (TME).^4^ Macrophages can account for more than 50% of solid tumors and play an important role in cancer progression.^57^^,^^58^ The high permeability of TAMs is associated with poor prognosis.^59^, ^60^, ^61^, ^62^, ^63^ They are usually classified as either an antitumor phenotype (M1-like) or a tumor-friendly phenotype (M2-like). Most TAMs exhibit an M2 phenotype that supports tumor growth, immune escape, and metastasis^4^ and promotes therapeutic resistance through various mechanisms.^57^^,^^60^^,^^64^, ^65^, ^66^, ^67^ M2 TAMs can also counteract the effect of cytotoxic agents on cancer cells through the secretion of survival signals and cathepsins.^64^^,^^65^ IL-4 and IL-13 are the main cytokines that polarize macrophages into the M2 subpopulation.^4^ Therefore, blocking the IL-4/IL-13 pathway may have anticancer effects. However, the tumor microenvironment is complex and dynamic and cannot be fully simulated by *in vitro* models. In an animal model of prostate cancer, drug inhibition of IL4Rα did not affect tumor growth.^4^ Therefore, further *in vitro* and *in vivo* tests are needed to evaluate the effect of targeting the IL4/IL13 pathway in different tumors.

### Roles of immunoglobulin G4 in tumors

Dupilumab is a fully human monoclonal antibody of the immunoglobulin G4 (IgG4) subclass. IgG4 antibody have a unique affinity profile for Fc gamma receptors (FcγRs) and support phenotypical macrophage changes towards an M2b-like state.^68^ Macrophages express FcγRIIa which is involved in antibody-dependent cellular phagocytosis (ADCP).^69^ Since IgG4 has a low affinity for FcγRIIa and a higher affinity for inhibitory FcγRIIb than for other IgG subclasses and only acts as an inhibitory effect when other FcγRs are co-involved,^70^ it is possible that IgG4 may dampen FcγR immune activation by co-engaging FcγRIIb together with the engagement of any other FcγRs by antigen-specific IgG1. Furthermore, since IgG4 is not able to trigger complement-dependent cytotoxicity (CDC),^71^ any tumor specific IgG4 antibody competing with tumor specific IgG1 antibody indirectly reduces IgG1-mediated CDC. Therefore, IgG4 is a key to immune tolerance in cancer. Previous study has found that IgG4 inhibited IFNγ signaling via FcγRI, and favoring an M2b-like phenotype,^72^ which plays a role in the formation of CTCL by secreting various chemokines,^52^ such as CCL-1, IL-10, and IL-6.^68^ CCL1 secretion is critical to maintain the M2b phenotype in mice and humans,^73^ while IL-10 has been found to impair the differentiation of infiltrated monocytes into mature dendritic cells (DCs), thereby compromising the competent host anti-tumor immune response.^74^ Through the analysis of CTCL patients and animal experiments, it has been proved that M2-like phenotype macrophages play an important role in the tumorigenesis of CTCL, and the depletion of macrophages inhibits tumor growth in a mouse model.^75^ In conclusion, as an IgG4 monoclonal antibody, dupilumab may promote tumor immune escape by affecting macrophage polarization and cytokine secretion.

### Tumor, AD, and biological treatment implications

#### In case of AD overlap with tumor

In our study, most AD patients with tumors showed improvement in AD symptoms, tumor stabilization, or regression after treatment with dupilumab. Only a few patients showed tumor progression, which was mainly MM and CTCL. Combined with the above analysis, we suggest that in most cases, dupilumab has no effect on tumor progression or even prevents tumor progression by blocking the IL-4/IL-13 pathway and/or inhibiting the transformation of TAMs to the M2 phenotype. However, dupilumab may promote tumor progression by blocking IL-13Rα1 and then increasing IL-13 binding to IL13Rα2, thus promoting tumor progression in some tumors, especially CTCLs. The effect of dupilumab on tumors may be determined by whether IL4/IL13 signaling plays a dominant role in tumors. Different tumors have distinctive signaling pathways with pro-tumor and tumor-suppressive roles. Furthermore, advanced CTCLs are aggressive. It is unclear whether dupilumab treatment worsens the disease or whether it is the natural course of the disease's progression. Due to the limited sample size, more observations and studies are needed to determine the effect of dupilumab on primary tumors.

#### Biologic treatments in AD may induce tumor

Some patients with dupilumab treatment developed new tumors, including CTCL in the majority, as well as other skin tumors, hematological tumors, and solid tumors such as bladder cancer. For individual patients with new solid tumors such as bladder cancer, the authors noted that there was no significant correlation between the occurrence of tumors and the use of dupilumab.^39^ However, we should pay special attention to CTCL. A group of patients with an initial diagnosis of AD confirmed pathologically atypical lymphocyte infiltration following dupilumab treatment.^44^ Over an average of 9.8 months after dupilumab treatment, the density, distribution pattern, and composition of lymphatic infiltrates gradually changed from reactive to neoplastic patterns.^44^ Previous data have supported the progressive development of CTCL in the context of chronic inflammatory processes such as AD driven by exogenous and endogenous factors.^76^ Therefore, dupilumab may be a potential trigger for the initial progression of benign lymphocyte tissue infiltration, leading to clonal expansion of T lymphocytes and subsequent malignant transformation. Although this study was limited by its retrospective design and sample size, it reminds us that careful clinical, histopathological, and immunohistochemical evaluation should be performed before and during the treatment of refractory AD and that continuous skin biopsy is necessary.

#### In case of misdiagnosis with AD

As described above, individual patients were misdiagnosed with AD and were given dupilumab treatment. After the treatment, the clinical symptoms worsened, and the diagnosis of CTCL was confirmed by multiple biopsies and other relevant examinations. Because CTCL can simulate multiple clinical manifestations of inflammatory skin diseases, especially in the early stages, repeated biopsies are required to assist in a definitive diagnosis. The prognosis and treatment regimens of AD and CTCL are largely different. Therefore, it is necessary to perform multiple or repeated pathological biopsies on refractory AD patients or AD patients with atypical skin lesions to determine whether there is a diagnosis of CTCL.

We summarized the literature on tumors in the setting of dupilumab use and came up with the following conclusions: dupilumab is theoretically safe in patients with concomitant tumors, but a small number of patients, especially those with CTCLs, developed progression of the primary tumor after treatment. Although a clear correlation between dupilumab therapy and tumor progression cannot be demonstrated, dupilumab does not have a definite effect on preventing tumor progression. In clinical studies of dupilumab for other diseases, such as asthma, chronic rhinosinusitis with nasal polyps, and eosinophilic oesophagitis, a small number of patients had serious adverse events related to neoplasms, which was ultimately determined without significant relationship to dupilumab treatment.^77^, ^78^, ^79^, ^80^ Due to the small sample size of relevant studies and the characteristics of the advanced malignant behavior of CTCL itself, it is still uncertain whether dupilumab causes tumor progression, and it is necessary to pay close attention to tumor changes during treatment. In addition, early CTCL is easily misdiagnosed as AD, which has a distinctive prognosis and treatment, so it is necessary to make a clear and definitive diagnosis. In a cross-sectional study of dupilumab-associated MF, the more advanced disease stage at the time of MF diagnosis during dupilumab use, the shorter the treatment duration to MF onset. In addition, older age and male sex seem to have a higher risk of advanced MF.^81^ Therefore, close monitoring of elderly men and late-stage MF patients with serial biopsies and close observation of clinical changes may be warranted. If patients show refractory or atypical lesions of AD, the possibility of CTCL should be considered. We suggest that biopsy criteria should be lowered before the application of biologics, and close follow-up should be conducted during treatment to evaluate the presence of CTCL based on clinical manifestations, pathological biopsies, TCR rearrangement, and immunological tests. Once a diagnosis of CTCL is established, caution is recommended to discontinue dupilumab and aggressively pursue lymphoma-related therapy. Thus, a multidisciplinary committee with oncologists is recommended to jointly assess the patient's condition and guide more precise treatment.

In conclusion, dupilumab, as the first monoclonal antibody approved for the treatment of moderate to severe AD, makes a significant contribution to improving the quality of life of patients with AD and AD-like symptoms. However, its safety and efficacy in the context of cancer remain unclear. In this study, we conclude that tumors are not an absolute contraindication for dupilumab, but careful evaluation before and during treatment is warranted. Limitations of this review include small sample size, incomplete clinical data of patients, short mean follow-up time, and inability to know the long-term prognosis of patients. More clinical reports and mechanistic studies are needed to clarify the safety and efficacy of dupilumab in the tumor setting.

## Conclusion

Here, we summarize the use of dupilumab in the tumor setting and cases of new tumors after dupilumab treatment in the literature and describe the demographics, clinical characteristics, therapeutic responses, and clinical outcomes of these patients. Based on these findings, we conclude that dupilumab is not an absolute contraindication for tumor use, but also does not have a definite tumor therapeutic effect. We recommend early and repeated testing in refractory AD patients with atypical lesions to identify the possibility of concomitant tumors, especially CTCLs. We suggest that the criteria for biopsy may be lowered appropriately and note that negative or ambiguous results do not preclude a diagnosis of CTCL. Dupilumab can be discontinued out of caution when the tumor is detected and active tumor-related therapy is initiated. It is necessary to follow up closely before and during treatment and to monitor the occurrence and development of tumors.

## Abbreviations

AD, atopic dermatitis; CTCL, Cutaneous T-cell lymphoma; IL, interleukin; IL-4Rα, interleukin-4 receptor alpha subunit; Th2, T-helper 2; MF, mycosis fungoides; TAM, tumor-associated macrophage; PRISMA, Preferred Reporting Items for Systematic Reviews and Meta-Analyses; ILC2, group 2 innate lymphoid cell; STAT4, signal transducer and activator of transcription 4; TME, tumor microenvironment; CLL, chronic lymphocytic leukemia; Dpl, dupilumab treatment; EDHM, eosinophilic dermatosis of hematologic malignancy; MM, multiple myeloma; MTX, methotrexate; SS, Sézary syndrome.

## Authors’ consent for publication

All authors give their consent to the publication of this manuscript.

## Data availability statement

Data is available on request from the authors.

## Ethics statement

Not applicable.

## Funding sources

This study was supported by the Guangzhou Science and Technology Program Basic Research Program - City School (Hospital) Jointly Funded Project - Yat-Sen Youth Medical Talent Plan (Grant No. 2024A03J0917); National Nature Science Foundation of China (82203089).

## Declaration of competing interest

None.
