# Supplementary material for: Tumors in the setting of dupilumab use: A review of the literature
Source: World Allergy Organ J. 2024 Dec 11;18(1):101006. doi: 10.1016/j.waojou.2024.101006 (PMC11697539; doi:10.1016/j.waojou.2024.101006)
Supplement: Multimedia component 1 [file mmc1.docx]

**Table S1. Detail clinical characteristics of patients with concomitant or newly emerging tumors treated with dupilumab**

| **Case No.** | **Sex/age (y)** | **Clinical diagnosis before dupilumab treatment** | **Treatment before dupilumab treatment** | **Time of the tumor diagnosed** | **Tumor type / TNM classification & stage** | **Dupilumab treatment period (month)** | **Change of the tumor after dupilumab treatment** | **Change of skin lesion after dupilumab treatment** | **Final diagnosis** | **Outcome** | **Reference** |
| --- | --- | --- | --- | --- | --- | --- | --- | --- | --- | --- | --- |
| **Cutaneous T-cell lymphoma** | | | | | | | | | | | |
| 1 | M/64 | Presumed AD (retrospectively diagnosed as CTCL not otherwise specified, stage Ib) | Azathioprine, topical corticosteroids, prednisone | After Dpl (retrospective diagnosis) | CTCL / stage IIIa | 8 | Progressive | Decrease in body surface area and pruritus in the first month; then palmoplantar desquamation, severe skin burning/pruritus, development of erythroderma, and *S. aureus* impetiginization | CTCL-not otherwise specified | Improvement with radiation therapy, bexarotene, and interferon-α | [8] |
| 2 | M/72 | Presumed AD | MTX | After Dpl | MF / stage Ib | 4 | - | Decrease in body surface area and pruritus in the first month; then thickening of plaques with superimposed papules | MF | Improvement with phototherapy and topical corticosteroids | [8] |
| 3 | F/59 | Presumed AD | Mycophenolate mofetil, cyclosporine A, topical corticosteroids, omalizumab | After Dpl | MF / stage Ia | 27 | - | Decrease in body surface area and pruritus in the first 8 months; then enlargement of facial plaque and onset of fatigue and weight loss | MF | Continuing Dpl at longer intervals, given patient preference and atopic benefits | [8] |
| 4 | F/40 | Presumed AD | MTX, topical corticosteroids | After Dpl | MF / stage IIIa | 15 | - | Decrease in body surface area in the first 4 months; then development of erythroderma and blepharoconjunctivitis, worsening pruritus | MF | Improvement with prednisone taper, triamcinolone, MTX, and phototherapy | [8] |
| 5 | M/67 | MF, stage IIIb | Photochemotherapy, MTX, bexarotene, IFN-α, extracorporeal photopheresis, romidepsin, tipifarnib, prednisone, everolimus, cyclophosphamide, Olaparib, methylprednisolone, doxorubicin | Before Dpl | MF / stage IVa | 3 | Progressive | Decrease in body surface area and pruritus in first 2 months, then palmoplantar desquamation, increase in body surface area to 100%, lymphadenopathy, worsening pruritus, fatigue, *S. aureus* impetiginization | MF | Progressive disease and death | [8] |
| 6 | M/58 | MF, stage IIa | Bexarotene, topical corticosteroids, and mechlorethamine | Before Dpl | MF / stage IVa | 3 | Progressive | Improved asthma and mild decrease in body surface area in the first 1.75 months; then increase in body surface area, lymphadenopathy, worsening pruritus, and fatigue | MF / SS | Death | [8] |
| 7 | F/77 | MF, stage Ib | Topical clobetasol, prednisone, excimer laser | Before Dpl | MF / stage IVa | 3 | Progressive | Development of erythroderma and lymphadenopathy, worsening pruritus | MF / SS | Endocarditis, partial response to romidepsin | [8] |
| 8 | F/66 | Chronic dermatitis | Topical and systemic corticosteroids, phototherapy, MTX, oral antihistamines, dapsone, gabapentin, adalimumab, mycophenolate mofetil | After Dpl | MF / T2aN2M0 and stage IIa | 1.2 | - | - | MF | Followed up for 8 months | [4] |
| 9 | F/65 | Chronic AD associated with idiopathic eosinophilia | Topical and systemic corticosteroids, phototherapy, hydroxyurea | After Dpl | MF / T2An0m0 and stage Ib | 2.5 | - | - | MF | - | [4] |
| 10 | M/74 | AD | Topical and systemic corticosteroids, aprelimast, pimecrolimus | After Dpl | MF / T4N0M0 and stage III | 24 | - | - | MF | - | [4] |
| 11 | M/73 | Chronic dermatitis | Topical and systemic corticosteroids, MTX, cyclosporine A, mycophenolate mofetil, azathioprine, omalizumab, antihistamines | After Dpl | MF / T4N0M0 and stage III | 14 | - | - | MF | Followed up for 16 months | [4] |
| 12 | M/74 | Chronic dermatitis | Topical and systemic corticosteroids, phototherapy, MTX, mycophenolate mofetil, secukinumab, acitretin | After Dpl | MF / T2bN1bM0 and stage IIa | 1 | - | - | MF | - | [4] |
| 13 | F/44 | AD | Topical corticosteroids, MTX | After Dpl | MF / 40N3M0 and stage IV | 12 | - | - | MF | Death | [4] |
| 14 | M/27 | AD and allergic contact dermatitis | Topical and systemic corticosteroids | After Dpl | MF / T2An0m0 and stage Ib | 14 | - | - | MF | Followed up for 5 months | [4] |
| 15 | M/58 | AD, maxillary sinus carcinoma | Topical corticosteroids, chemoradiotherapy | Before Dpl (maxillary sinus carcinoma);  after Dpl (MF) | Maxillary sinus carcinoma, MF | 1 | - | Slight improvement of AD followed by exacerbation of lesions | MF, maxillary sinus carcinoma | Discontinuation of Dpl, initiation of MF treatment | [14] |
| 16 | F/51 | Presumed AD | Topical and systemic corticosteroids, azathioprine | After Dpl | MF | - | - | Slight improvement of pruritus, worsening skin lesions with tumor appearance | MF | Discontinuation of Dpl, treatment of acitretin and psoralen plus ultraviolet A with partial response | [49] |
| 17 | M/43 | AD | Topical and systemic corticosteroids, | After Dpl | MF / T2bN3M0B1b and stage IVa2 | 2 | - | Slight improvement in pruritus, worsening skin eruption | AD, MF | Discontinuation of Dpl, progressive MF | [38] |
| 18 | F/37 | AD | psoralen plus ultraviolet A, topical tacrolimus and corticosteroids, MTX, cyclosporine A | After Dpl | SS | 2 | - | No response | SS | Discontinuation of Dpl, change to mogamulizumab with favorable response | [21] |
| 19 | M/55 | AD, MF | - | Before Dpl | MF | 4 | Partial remission | Improvement in pruritus | AD, MF | Favorable outcome at 4 months of follow-up | [21] |
| 20 | F/48 | AD | - | After Dpl | SS / T4N2M0B2 and stage IVa1 | 2 | - | No response | SS | Discontinuation of Dpl, change to topical corticosteroids, phototherapy, and systemic vorinostat, with good response | [43] |
| 21 | F/74 | SS | Phototherapy, topical and systemic corticosteroids, cyclosporine A, psoralen plus ultraviolet A, extracorporeal photopheresis, INFα-2a | Before Dpl | SS / pT4N1B2M0 and stage IVa1 | 11 | Reduction in size of internal lymph nodes, increase of malignant T cells in blood | Marked improvement of skin lesions, pruritus, and quality of life | SS | Stable | [29] |
| 22 | F/50 | AD | Topical corticosteroids, cyclosporine A, MTX | After Dpl | Anaplastic large-cell lymphoma/ | 5 | Progressive | Complete response of AD, the occurrence of a new painful, erythematous, and ulcerated plaque (5 cm) on the right breast | Anaplastic large-cell lymphoma | Discontinuation of Dpl, change to polychemotherapy, and death | [45] |
| 23 | F/47 | AD | - | After Dpl | MF | Once | - | Development of flat nodules on cheeks | MF | Discontinuation of Dpl, change to a combination treatment of phototherapy and bexarotene, with a favorable outcome | [40] |
| 24 | M/64 | AD | Topical corticosteroids, UV light therapy | After Dpl | SS | - | - | Worsening skin lesions: erythrodermic rash covering 95% of the body | SS | Discontinuation of Dpl, change to bexarotene, and extracorporeal photopheresis | [41] |
| 25 | M/60 | AD (retrospectively diagnosed as MF) | - | After Dpl (retrospective diagnosis) | MF / stage IV | 4+ | - | Progression of skin lesions | MF | Change to chemotherapy and death | [22] |
| 26 | M/68 | AD, CTCL | Bexarotene, INFα-2b, INFγ-1b, total skin electron beam therapy, phototherapy, extracorporeal photopheresis, topical corticosteroids | Before Dpl | CTCL / stage IVa1 | 4 | Overall slight reduction | Improvement in pruritus and skin lesions | AD, CTCL | Followed up for 6 months | [12] |
| 27 | M/61 | Nonspecific eczematous dermatitis | Topical corticosteroids, phototherapy, prednisone | After Dpl | MF | - | - | - | MF | - | [46] |
| 28 | M/52 | Suspicion of psoriasis | Topical corticosteroids, injectable biologics, prednisone | After-Dpl | MF | 1+ | - | Initially improvement, then no response | MF | - | [46] |
| 29 | F/60 | Eczematous dermatitis | Topical corticosteroids, phototherapy, guselkumab | After Dpl | MF | 0.5 | - | No response | MF | - | [46] |
| 30 | F/48 | AD | MTX, UV light therapy | After Dpl | MF / stage Ib | 5 | - | No response | MF | - | [36] |
| 31 | M/55 | AD, oropharyngeal cancer | Topical corticosteroids, UV light therapy | Before Dpl (oropharyngeal cancer); after Dpl (MF) | Oropharyngeal cancer; MF / stage Ib | 6 | - | No response | MF | - | [36] |
| 32 | F/40 | AD, eosinophilic asthma, chronic eosinophilia | MTX | After Dpl | MF | 5 | - | Initially improvement, then progressive disease | MF | - | [47] |
| 33 | F/75 | CTCL, malignant melanoma in situ | Whole-body electron beam therapy, IFN-α, topical nitrogen mustard, topical corticosteroids, belinostat, gemcitabine, local retinoids, liposomal doxorubicin, surgery | Before Dpl | CTCL, malignant melanoma in situ | 41 | - | Improvement in Itching significantly | CTCL, malignant melanoma in situ | Stable | [17] |
| 34 | F/79 | AD | Topical corticosteroids | After Dpl (retrospective diagnosis) | MF/stage IA | - | - | Improvement in body lesions, but the facial rash progressed | MF/stage IA | - | [13] |
| **Other skin tumors** | | | | | | | | | | | |
| 35 | F/22 | AD, melanoma | Topical mometosone, crisaborole, mirtazapine | Before Dpl | Melanoma | 18 | Remission | Improvement of pruritus | AD, melanoma | Improvement of AD | [35] |
| 36 | M/43 | AD, HIV infection, squamous cell carcinoma, *S. aureus*  impetigizination | Chemotherapy, radiation, clobetasol, triamcinolone, pimecrolimus, phototherapy | Before Dpl | Squamous cell carcinoma | 9 | Remission | Improvement of pruritus | AD, HIV infection, squamous cell carcinoma, *S. aureus* impetigizination | Improvement of AD | [35] |
| 33 | F/75 | CTCL, malignant melanoma in situ | Whole-body electron beam therapy, IFN-α, topical nitrogen mustard, topical corticosteroids, belinostat, gemcitabine, local retinoids, liposomal doxorubicin, surgery | Before Dpl | CTCL, malignant melanoma in situ | 41 | - | Improvement in Itching significantly | CTCL, malignant melanoma in situ | Stable | [17] |
| 37 | M/32 | Melanoma | Nivolumab, surgery, ipilimumab, antihistamines, corticosteroids | Before Dpl | Melanoma | 10 | - | Improvement in pruritus | Melanoma | Recurrence of pruritic symptoms after discontinuation of dupilumab therapy | [17] |
| 38 | M/74 | CLL, recurrent invasive cutaneous squamous cell carcinoma, metastatic melanoma | Cemiplimab, topical steroids | Before Dpl | CLL, recurrent invasive cutaneous squamous cell carcinoma, metastatic melanoma | 5 | SCC complete response, metastatic melanoma partial response, but has persistently enlarged lymph nodes | Maintained eczema and pruritus control | CLL, recurrent invasive cutaneous squamous cell carcinoma, metastatic melanoma, immune-related vitiligo | - | [15] |
| 39 | M/78 | Melanoma/stage IIIC, bullous pemphigoid | Nivolumab, clobetasol, prednisone | Before Dpl | Melanoma/stage IIIC | - | No evidence of melanoma recurrence. | Pruritus was well controlled with no new erosions or bullae | Melanoma/stage IIIC, bullous pemphigoid | - | [15] |
| 40 | M/66 | Melanoma/pT3bn1a | Nivolumab, oral methylprednisolone, topical mometasone, oral prednisone | Before Dpl | Melanoma/pT3bn1a | - | Stable | Remission of dermatitis | Melanoma/pT3bn1a | Followed up for 9 months, and the patient was free from disease | [26] |
| 41 | M/65 | Osseous metastatic angiosarcoma, bullous pemphigoid | Chemotherapy with gemcitabine and dacarbazine, ipilimumab, nivolumab, prednisolone | Before Dpl | Osseous metastatic angiosarcoma | - | Stable | No recurrence of bullous pemphigoid | Osseous metastatic angiosarcoma, bullous pemphigoid | Continue effective treatment of tumor disease | [32] |
| 42 | M/75 | Melanom/pT2a, bladder cancer, high grade urothelial transitional cell carcinoma/pT2G3 | Surgery, chemoradiotherapy, nivolumab, topical steroids, antihistamines | Before Dpl | Melanom/pT2a, bladder cancer, high grade urothelial transitional cell carcinoma/pT2G3 | - | Reduction in size of previous enlarged nodule | No recurrence of AD | Melanom/pT2a, bladder cancer, high grade urothelial transitional cell carcinoma/pT2G3 | Stable | [30] |
| **Hematological tumor** | | | | | | | | | | | |
| 43 | M/59 | CLL, stage II | Ibrutinib, cephalexin, triamcinolone, prednisone | Before Dpl | CLL/ stage II | 6.5 | - | Clearance of skin lesions after 3 doses of Dpl | CLL, EDHM | Death due to hypoxic respiratory failure | [33] |
| 44 | M/81 | CLL, leukemia cutis, EDHM | Acyclovir, valacyclovir, prednisone, rituximab, chlorambucil | Before Dpl | CLL | 6+ | - | Lesions resolved after 2 injections of Dpl | CLL, leukemia cutis, EDHM | Clearance of skin lesions | [25] |
| 45 | F/25 | AD, Hodgkin’s lymphoma | Autologous stem cell transplant | Before Dpl | Hodgkin’s lymphoma / IIb | - | - | - | AD, Hodgkin’s lymphoma | Followed up for >12 months | [10] |
| 46 | -/- | CLL, EDHM | - | Before Dpl | CLL | - | - | - | CLL, EDHM | Marked improvement | [24] |
| 47 | -/- | CLL, EDHM | - | Before Dpl | CLL | - | - | - | CLL, EDHM | Partial improvement | [24] |
| 48 | -/- | CLL, EDHM | - | Before Dpl | CLL | - | - | - | CLL, EDHM | Stable | [24] |
| 49 | -/- | CLL, EDHM | - | Before Dpl | CLL | - | - | - | CLL, EDHM | Worsening | [24] |
| 50 | -/- | Follicular lymphoma, EDHM | - | Before Dpl | Follicular lymphoma | - | - | - | Follicular lymphoma, EDHM | Worsening | [24] |
| 51 | -/- | CLL, EDHM | - | Before Dpl | CLL | - | - | - | CLL, EDHM | Worsening | [24] |
| 52 | F/50s | Small lymphocytic lymphoma, EDHM | Rituximab, bendamustine, prednisone | Before Dpl | Small lymphocytic lymphoma | - | Complete remission | Improvement of pruritus and skin lesions | Small lymphocytic lymphoma, EDHM | Followed up for 20 months, without relapse | [18] |
| 53 | F/56 | AD, non-Hodgkin’s lymphoma | Rituximab, topical corticosteroids, tacrolimus, antihistamines | Before Dpl | Non-Hodgkin’s lymphoma/stage IIa | 5 | Stable | Improvement of pruritus and skin lesions | AD, non-Hodgkin’s lymphoma | Improvement of AD | [31] |
| 54 | F/67 | IgG Kappa MM | Lenalidomide, systemic corticosteroids | Before Dpl | MM / stage III | - | Progressive | Rash resolved | MM | Switched to the next line of therapy due to progressive disease | [19] |
| 55 | M/62 | MM | Lenalidomide, systemic corticosteroids | Before Dpl | MM / stage III | - | - | Rash resolved | MM | Stable | [19] |
| 56 | M/75 | IgA Kappa MM | Lenalidomide, systemic corticosteroids | Before Dpl | MM | - | - | Rash resolved | MM | Very good partial response | [19] |
| 57 | M/62 | IgG Kappa MM | Lenalidomide, systemic corticosteroids, daratumumab | Before Dpl | MM/stage III | - | - | Rash resolved and then recurred | MM | Continuation of Dpl with excellent control results | [19] |
| 58 | M/58 | IgG Lambda MM | Lenalidomide, systemic corticosteroids, daratumumab, carfilzomib | Before Dpl | MM/stage II | - | - | Rash resolved | MM | Stable | [19] |
| 59 | M/54 | IgG Kappa MM | Lenalidomide, systemic corticosteroids | Before Dpl | MM/stage II | - | Progressive | Rash resolved | MM | Switched to the next line of therapy due to progressive disease | [19] |
| 60 | M/65 | IgG Kappa MM | Melphalan, autologous stem cell transplant, lenalidomide, topical corticosteroids, phototherapy | Before Dpl | MM/stage II | 19 | Complete response for 12 months; then stable disease for 7 months; progression of disease after discontinuation of Dpl | Rash resolved | MM | Switched to chemotherapy and other MM-specific therapies | [20] |
| 61 | M/70 | IgG Kappa MM | Melphalan, autologous stem cell transplant, lenalidomide | Before Dpl | MM/stage I | 17 | Complete response for 15 months, then a progression of disease | Rash resolved | MM | Switched to daratumumab and immunomodulatory drugs | [20] |
| 62 | M/66 | IgG Kappa MM | lenalidomide, daratumumab,  and bortezomib | Before Dpl | MM/stage I | 4 | Remaining in very good partial response | Rash resolved | MM | Switched to topical treatment | [20] |
| 63 | M/38 | AD | Topical corticosteroids | After Dpl | Nodal anaplastic large cell lymphoma with lymphomatoid papulosis | 12 | - | Development of steroid-resistant disseminated papules | AD, nodal anaplastic large cell lymphoma with lymphomatoid papulosis | Switched to brentuximab and vedotin; disappearance of crusted nodules and disseminated red papules | [48] |
| 64 | M/47 | AD | - | After Dpl | Discordant lymphomas of  Hodgkin lymphoma and peripheral T-cell lymphoma | - | - | - | AD, discordant lymphomas of  Hodgkin lymphoma and peripheral T-cell lymphoma-not otherwise specified | - | [42] |
| 65 | M/70 | Presumed AD | - | After Dpl | Peripheral T-cell lymphoma, NOS | 2 | Lymphadenopathy and rash improved with THP-COP, but his course was complicated by recurrent neutropenic fever | Rash initially improved to a moderate extent but aggravated afterward | Peripheral T-cell lymphoma, NOS | The patient declined further chemotherapy and pursued palliative care | [37] |
| 38 | M/74 | CLL, recurrent invasive cutaneous squamous cell carcinoma, metastatic melanoma | Cemiplimab, topical steroids | Before Dpl | CLL, recurrent invasive cutaneous squamous cell carcinoma, metastatic melanoma | 5 | SCC complete response, metastatic melanoma partial response, but has persistently enlarged lymph nodes | Maintained eczema and pruritus control | CLL, recurrent invasive cutaneous squamous cell carcinoma, metastatic melanoma, immune-related vitiligo | - | [15] |
| **Solid tumor** | | | | | | | | | | | |
| 66 | M/82 | Breast cancer | Pembrolizumab, oral methylprednisolone, topical clobetasol | Before Dpl | Breast cancer | - | - | Remission of dermatitis | Breast cancer | Followed up for 9 months, and the patient was free from dermatitis | [26] |
| 67 | F/56 | AD, infiltrating ductal breast cancer | - | Before Dpl | Infiltrating ductal breast cancer / pT1c-pN0 | - | - | - | AD, infiltrating ductal breast cancer | / | [10] |
| 68 | F/64 | AD, lobular breast cancer | - | Before Dpl | Lobular breast cancer in situ; infiltrating lobular breast cancer / pT2pN0 after 2 years | - | - | - | AD, infiltrating lobular breast cancer | Followed up for >12 months | [10] |
| 69 | M/77 | AD, colon adenocarcinoma | - | Before Dpl | Colon adenocarcinoma / pT1N0, and T2cN0 after one year | - | - | - | AD, colon adenocarcinoma | - | [10] |
| 70 | M/40 | AD, papillary thyroid carcinoma | - | Before Dpl | Papillary thyroid carcinoma / pT1bN0 | - | - | - | AD, papillary thyroid carcinoma | Followed up for >12 months | [10] |
| 71 | M/60 | AD, papillary urothelial bladder carcinoma | - | Before Dpl | Papillary urothelial bladder carcinoma / G3pT1 | - | - | - | AD, papillary urothelial bladder carcinoma | Followed up for >12 months | [10] |
| 73 | -/53 | AD, lung adenocarcinoma | - | Before Dpl | Lung adenocarcinoma / stage IV | - | - | - | AD, lung adenocarcinoma | - | [10] |
| 73 | M/32 | AD | - | After Dpl | Seminoma / pT2 | - | - | - | AD, seminoma | - | [10] |
| 74 | M/23 | AD | - | After Dpl | Embryonic carcinoma | - | - | - | AD, embryonic carcinoma | - | [10] |
| 75 | F/52 | AD, heart failure, kidney tumor | Topical and systemic corticosteroids, phototherapy | Before Dpl | Renal oncocytoma (Grawitz tumor?) | 12 | - | Improvement of pruritus and skin lesions | AD, heart failure, kidney tumor | Followed up for 12 months, AD remaining but well controlled | [9] |
| 76 | F/30 | AD, type 1 neurofibromatosis | Systemic corticosteroids, cyclosporine A | Before Dpl | Type 1 neurofibromatosis | 18 | Improvement of type 1 neurofibromatosis | Improvement of AD | AD, type 1 neurofibromatosis | Stable | [23] |
| 77 | F/59 | AD, colorectal cancer, arterial hypertension, hypothyroidism | Surgery and chemotherapy | Before Dpl | Colorectal cancer/stage IIIb | 12+ | Remission | Improvement of pruritus | AD, colorectal cancer, arterial hypertension, hypothyroidism | Stable | [34] |
| 78 | F/46 | AD, colorectal cancer, kidney cancer, bronchial asthma | Surgery, chemotherapy, radiotherapy, topical corticosteroids, phototherapy | Before Dpl | Colorectal cancer / IIIa; kidney cancer / Ia | 30 | No tumor progression | Improvement of pruritus | AD, colorectal cancer, kidney cancer, bronchial asthma | Stable | [34] |
| 79 | M/56 | AD, Penile spinocellular carcinoma, prostate cancer, bronchial asthma | Surgery | Before Dpl | Penile spinocellular carcinoma, prostate cancer | 5 | No tumor recurrence | Improvement of pruritus | AD, Penile spinocellular carcinoma, prostate cancer, bronchial asthma | Stable | [34] |
| 80 | M/73 | Metastatic renal cell carcinoma, Grover disease, bullous pemphigoid | Ipilimumab, nivolumab, topical corticosteroids, prednisone | Before Dpl | Metastatic renal cell carcinoma | - | Full remission | Full remission | Metastatic renal cell carcinoma, Grover disease, bullous pemphigoid | - | [11] |
| 81 | M/46 | AD, giant condyloma acuminatum | Steroids, cyclosporine A | After Dpl | Bladder cancer / pTa | 4 | - | Improvement of pruritus | AD, giant condyloma acuminatum, bladder cancer | Discontinuation of Dpl | [39] |
| 82 | F/59 | AD, arterial hypertension, hypothyroidism, colorectal cancer | UVB phototherapy, nemolizumab, surgery, capecitabine | Before Dpl | Colorectal cancer/stage IIIb | 12+ | Stable | Improvement in pruritus and skin lesions | AD, arterial hypertension ,hypothyroidism, colorectal cancer | Stable | [34] |
| 83 | F/46 | AD, bronchial asthma, polyvalent allergy, colorectal cancer stage IIIa, carcinoma of right kidney stage Ia | Surgery, chemotherapy, radiotherapy, topical corticosteroids, narrowband UVB phototherapy | Before Dpl | Colorectal cancer/stage IIIa, carcinoma of right kidney/stage Ia | 30+ | No tumor recurrence | Improvement in pruritus and skin lesions | AD, bronchial asthma, polyvalent allergy, colorectal cancer stage IIIa, carcinoma of right kidney stage Ia | Stable | [34] |
| 84 | M/56 | AD, bronchial asthma, polyvalent allergy, penile spinocellular carcinoma, prostate cancer | Surgery, topical and systemic corticosteroids, narrowband UVB phototherapy | Before Dpl | Penile spinocellular carcinoma, prostate cancer | 5 | No tumor recurrence | Improvement in pruritus and skin lesions | AD, bronchial asthma, polyvalent allergy, penile spinocellular carcinoma, prostate cancer | Stable | [34] |
| 85 | F/68 | Rheumatoid arthritis, pancreatic adenocarcinoma | Adalimumab, hydroxychloroquine, intermittent prednisone, pembrolizumab, acitretin, topical halobetasol, prednisone | Before Dpl | Pancreatic adenocarcinoma | - | Stable | Improvement in rash and itch | Rheumatoid arthritis, pancreatic adenocarcinoma | Still being treated with pembrolizumab and dupilumab | [28] |
| 86 | M/72 | Metastatic clear cell renal cell carcinoma | Pembrolizumab, topical halobetasol, methotrexate | Before Dpl | Metastatic clear cell renal cell carcinoma | - | Stable | Rash cleared within 1 month | Metastatic clear cell renal cell carcinoma | Still being treated with pembrolizumab and dupilumab | [28] |
| 87 | F/74 | Metastatic lung adenocarcinoma, radiation-induced morphoea | Pembrolizumab, radiotherapy, intralesional triamcinolone, mupirocin ointment, tacrolimus ointment, gabapentin cream | Before Dpl | Metastatic lung adenocarcinoma | 12 | Stable | Improvement in pain and skin lesions | Metastatic lung adenocarcinoma, radiation-induced morphoea | Stable | [27] |
| 88 | M/73 | Prostate cancer | Surgery, radiation therapy, triptorelin, topical tacrolimus and moisturizer | Before Dpl | Prostate cancer | 27 | Stable | Itching completely resolved | Prostate cancer | Stable | [17] |
| 89 | F/70 | Recurrent high-grade serous endometrial carcinoma | Pembrolizumab, lenvatinib, lenvatinib, clobetasol, doxycycline, nicotinamide, oral prednisolone, mycophenolate mofetil | Before Dpl | Recurrent high-grade serous endometrial carcinoma | - | Growing peritoneal, liver, spleen, and lymph node metastases | - | Recurrent high-grade serous endometrial carcinoma | The patient decided to stop all cancer treatments and died in the palliative care service. | [15] |
| 90 | M/47 | AD, allergic keratoconjunctivitis, renal cell carcinoma | Topical steroids, narrow band UVB, oral corticosteroids, cyclosporin A, azathioprine, methotrexate, mycophenolate mofetil, surgery, stereotactic radiotherapy, ipilimumab, nivolumab, lenvatinib, belzutifan | Before Dpl | Renal cell carcinoma | 60 | Stable | Improvement in pruritus and skin lesions | AD, allergic keratoconjunctivitis, renal cell carcinoma | Stable | [16] |
| 42 | M/75 | Melanom/pT2a, bladder cancer, high grade urothelial transitional cell carcinoma/pT2G3 | Surgery, chemoradiotherapy, nivolumab, topical steroids, antihistamines | Before Dpl | Melanom/pT2a, bladder cancer, high grade urothelial transitional cell carcinoma/pT2G3 | - | Reduction in size of previous enlarged nodule | No recurrence of AD | Melanom/pT2a, bladder cancer, high grade urothelial transitional cell carcinoma/pT2G3 | Stable | [30] |

AD, atopic dermatitis; CLL, chronic lymphocytic leukemia; CTCL, Cutaneous T-cell lymphoma; Dpl, dupilumab treatment; EDHM, eosinophilic dermatosis of hematologic malignancy; MF, mycosis fungoides; MM, multiple myeloma; MTX, methotrexate; SS, Sézary syndrome; -, data not available
